# Rapid identification and isolation of patients with COVID-19 reduces the odds of transmission to hospital roommates

**DOI:** 10.1017/ash.2022.131

**Published:** 2022-05-16

**Authors:** Jessica Alban, Patrick Burke, Joanne Sitaras, Thomas Fraser

## Abstract

**Background:** The Cleveland Clinic Main Campus is a multispecialty academic medical center with 1,200 adult patient beds, 58% of which are double occupancy. Our facility relies on double-occupancy rooms to provide needed care during the COVID-19 pandemic. Inherently, double occupancy poses a greater risk of exposure to SARS-CoV-2 despite mitigation efforts. We investigated the incidence of postexposure SARS-CoV-2 infection in double-occupancy rooms and evaluated risk factors for viral transmission. **Methods:** Early in the observation period patients were tested for SARS-CoV-2 based on clinical suspicion. By June 2020, all admitted patients were tested. Symptomatic patients were admitted with pending tests under transmission-based precautions. Asymptomatic patients were managed with standard precautions including patients admitted to double-occupancy rooms. A double-occupancy exposure event was defined as an uninfected patient sharing a room with a patient positive for SARS-CoV-2. All patient exposures were tracked and evaluated by the infection prevention (IP) team. The IP prospective review of source patients included determination of lowest cycle threshold (Ct) value of first COVID-19 test, and whether their infection was hospital or community onset. Review of exposed patients included sex, age, and exposure time (in hours) to the source patient. Postexposure infection was defined as a positive test for SARS-CoV-2 in the exposed population within 14 days of the defined exposure event. We fit a multivariable logistic regression model to estimate the effect of exposure time on the odds of postexposure infection in susceptible roommates. **Results:** From March 15 to December 15, 2020, 172 susceptible patients were exposed to a roommate with COVID-19. Also, 28 exposed patients met our definition for postexposure infection (attack rate, 16%). The frequency of postexposure infection was higher in patients for whom the source was hospital-onset versus community-onset disease (25% vs 10%; *P* = .01) and when the source patient’s Ct value was below the median value of 21.1 (26% vs 11% p **Conclusions:** We identified a postexposure infection attack rate of 16% for double-occupancy patients in the first 9 months of the pandemic. Time exposed to source patient was significantly associated with infection. Our experience demonstrates the potential benefit of asymptomatic admission testing with expedited turnaround time to mitigate viral transmission between patients in double-occupancy rooms.

**Funding:** None

**Disclosures:** None

